# Design and Analysis of a Novel Flexure-Based Dynamically Tunable Nanopositioner

**DOI:** 10.3390/mi12020212

**Published:** 2021-02-19

**Authors:** Zeying Li, Pengbo Liu, Peng Yan

**Affiliations:** 1 School of Mechanical & Automotive Engineering, Qilu University of Technology (Shandong Academy of Sciences), Jinan 250353, China; zeying729@gmail.com; 2 Key Laboratory of High-Efficiency and Clean Mechanical Manufacturing, Ministry of Education, School of Mechanical Engineering, Shandong University, Jinan 250061, China; 3 Shenzhen Research Institute of Shandong University, Shenzhen 518057, China

**Keywords:** nanopositioning, compliant mechanism, tunable dynamics, variable stiffness, magnetorheological elastomers

## Abstract

Various tools, such as biomedical manipulators, optical aligners, and ultraprecision manufacturing tools, implement nanopositioners that must be dynamically tunable to satisfy the requirements of different working conditions. In this paper, we present the design and analysis of a flexure-based nanopositioner with dynamically tunable characteristics for the implementation of a high-performance servomechanism. The nanopositioner is composed of four flexure beams that are positioned in parallel and symmetric configurations sandwiched between magnetorheological elastomers (MREs). The properties of MREs impart dynamicity to the nanopositioner, allowing the workspace, stiffness, and damping characteristics in particular to be tuned under the action of an external magnetic field. By utilizing elastic beam theory and electromagnetic field coupling analysis, kinetostatic and dynamic models of the proposed nanopositioner were established to predict the variable stiffness property and dynamically tunable characteristics. The models were validated by performing a finite element analysis. Herein, it is shown that the proposed nanopositioner model can actively adjust the trade-offs between the working range, speed, and sustained load capability by changing the magnetic field. The proposed dynamic tuning method offers new insight into the design of flexure-based nanopositioners for real applications.

## 1. Introduction

Owing to the advantages of an ultrahigh accuracy and response speed, compliant mechanism-based nanopositioning systems [1,2] have emerged as one of the key enabling components in nanomeasurement, manipulation, and manufacturing instruments, such as atomic force microscopes [3], semiconductor lithography [4], and fast tool servo-assisted machining instruments [5]. However, their limited working range significantly limits the applicability of nanopositioning systems. Thus, the amount of research focused on the optimal design and control of nanopositioning systems has increased in recent decades [6,7,8,9,10]. 

The trade-off between motion stroke precision and mechanical bandwidth is known to be unavoidable; it exists because nanoscale-precision motion is realized through the elastic deformation of compliant mechanisms [11,12,13]. Various designs of nanopositioners with large workspaces (i.e., millimeter range) have been developed at the cost of a low stiffness [14,15,16], which reduced the load-carrying capability and response speed. Conversely, more flexibility also poses a significant challenge to high-precision nanopositioning control. Owing to the performance limitations of compliant mechanisms, research efforts have been devoted to exploring dynamic tuning technologies. Nanopositioning systems with tunable stiffness enable adjustment of the working range and mechanical bandwidth according to the operating conditions. Several controllable stiffness solutions, including cross-section shaping [17], the implementation of multilayer beams [18], preloading and boundary condition adjustment [19], layer jamming [20], and fluid-based approaches [21], have been applied in the design of compliant mechanisms and nanopositioning systems. However, the essential tuning mechanisms significantly affect the mechanical structure of the nanopositioning systems. Currently, the primary solution to the problem of variable stiffness is to change the elastic properties of the materials (e.g., the elastic modulus). Thus, magnetorheological elastomers (MREs), whose rheological and mechanical properties can be changed according to the externally applied magnetic field, provide a means of overcoming the disadvantages of the abovementioned methods [22,23,24].

With this as a motivation, we developed a novel dynamically tunable nanopositioner that exploited the advantages of MREs. In particular, the nanopositioner is driven by a voice coin motor (VCM) with a large actuation stroke. The sandwiched guiding mechanism composed of flexure beams and MREs was designed to be positioned on both sides of the central motion platform so that the variables affecting the mechanical properties, including the workspace, damping, and stiffness, as well as the dynamical behavior of the nanopositioner, could be actively controlled by an external magnetic field. Theoretical modeling and finite element analysis (FEA), as well as experiments were conducted to verify the proposed design concept.

The remainder of this paper is organized as follows. In Section 2, the mechanical structure of the developed dynamically tunable nanopositioner is described. In Section 3, kinetostatic and dynamic models are established to describe the variable stiffness and dynamically tunable properties. Section 4 outlines the FEA and experiment-based validation of the proposed design. Last, concluding remarks are provided in Section 5.

## 2. Motivation

The dynamic performance of a compliant mechanism-based nanopositioner is determined by its natural frequency, which is compromised by the workspace requirements. This means that a high natural frequency for compliant mechanisms can only be achieved at the expense of the working range. For example, a millimeter-range flexure-based nanopositioner typically performs at a resonant frequency of less than 100 Hz [14,15]. Various tools, such as biomedical manipulators, optical aligners, and ultraprecision manufacturing tools, require the dynamically tunable characteristics of nanopositioners to satisfy the requirements of different working conditions. Thus, for this study, we aimed to develop a dynamically tunable flexure-based nanopositioner that optimized the trade-off between the mechanical bandwidth (natural frequency) and working range (stroke).

## 3. Materials and Methods

### 3.1. Design Overview

**Figure 1:**
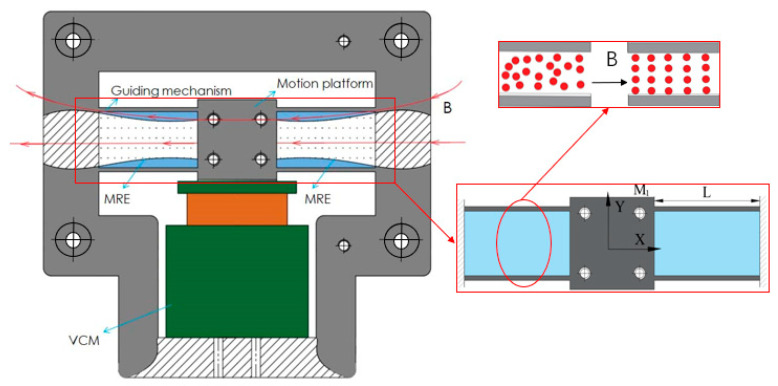
Schematic diagram of the proposed nanopositioner configuration.

As illustrated in Figure 1, a VCM isadopted as the actuator for the nanopositioner owing to its high resolution and fast response time. The moving coil is connected to the central motion platform, which is guided by a sandwiched guiding mechanism composed of flexure beams and MREs. Owing to the characteristics of a high longitudinal stiffness and low transverse stiffness as well as its ability to ensure a low stress concentration in the flexure beams, the nanopositioner can achieve large strokes and excellent robustness against parasitic motion. The rheological and mechanical properties of MREs, particularly the elastic modulus, can be changed according to the external magnetic field. Furthermore, the stiffness of the guiding mechanism, as well as the dynamic performance of the nanopositioner, can be dynamically adjusted by controlling the magnetic field. Note that due to the temperature-dependent mechanical properties of MREs, the magnetic-induced modulus of the MREs decreases with the heat generation from the MREs in the presence of the magnetic field, which leads to significant adverse effects on the dynamical performance of the nanopositioner. For real applications, water-cooling should be considered by designing channel structures inside the compliant mechanisms.

### 3.2. Kinetostatic Model of Guiding Mechanism

This section describes the kinetostatic model of the developed nanopositioner, which is constructed by applying the elastic beam theory and performing an MRE electromagnetic field coupling analysis under the condition of an accurate prediction of the variable stiffness. According to Lagrange’s equation, the dynamic model of the nanopositioner can then be derived by incorporating the action of a magnetic field.

#### 3.2.1. Deformation of the Flexure Beam

**Figure 2:**
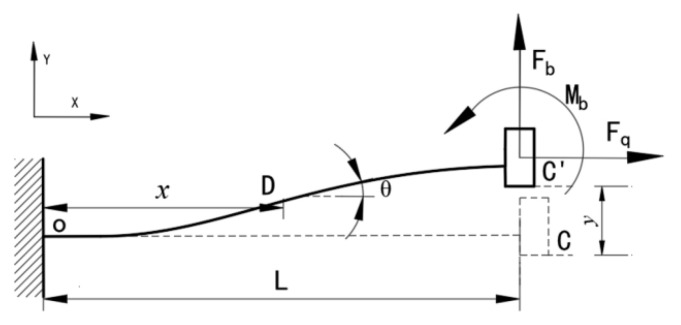
Analysis of the mechanics of a guided flexure beam.

As depicted in Figure 1, the guiding mechanism is composed of four flexure beams sandwiched between the MREs. First, we will describe the analysis procedure for the flexure beams. Owing to the symmetric structure and identical working conditions, only one flexure beam needs to be analyzed to establish the mathematical model. Because of the slender structure, we disregard the shear deflections of the flexure beam. Note that the transverse deformations of the flexure beams are an order of magnitude less than the beam length; this means that the axial stretch deformations of the flexure beams can be ignored. Accordingly, we perform a mechanical analysis of one flexure beam, as illustrated in Figure 2, where $F_{b}$ and $M_{b}$ are the transverse force and bending moment applied at the endpoint, respectively. According to the Euler–Bernoulli equation, we have:

(1) $$M\left(x\right)=EI\frac{d\theta }{ds}=EI\frac{d^{2}y}{dx^{2}}=M_{b}+F_{b}\left(L-x\right)$$

where M(x) is the equivalent bending moment applied at the arbitrary cross section, *x* is the length along the undeflected beam axis, *y* is the transverse deflection, *θ* is the angular deflection, dθ/ds is the rate of change of angular deflection along the beam, E is the elastic modulus, $I={\left(b_{b}t_{b}\right)}^{3}/12$ is the moment of inertia, and $b_{b}$, $t_{b}$, and L are the width, thickness, and length of the beam, respectively.

At the fixed end, the rotation and deflection are zero (i.e., θ(0)=0, y(0)=0). At the guiding end, the rotational angle is zero (i.e., θ(L)=0). These boundary conditions can then be applied to solve Equation (1) and obtain the deflection equation for the flexure beam, as follows:

(2) $$y(x)=\frac{F_{b}x^{2}\left(3L-2x\right)}{12EI}$$

Accordingly, we derive the guiding displacement δ as follows:

(3) $$\delta =y(L)=\frac{F_{b}L^{3}}{12EI}$$

#### 3.2.2. Analytical Model of MREs

**Figure 3:**
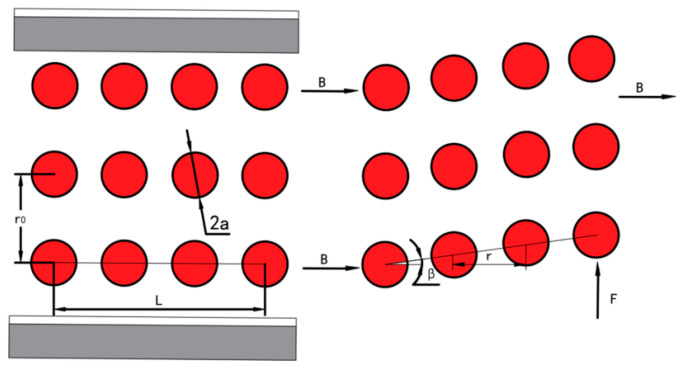
Predeformed and postdeformed structures of chains in the elastomer.

Next, we describe the procedure for modeling MREs, which are composed of magnetic particles and polymer bodies. Because of the magnetic dipole interactions that occur between particles, the shear modulus of MREs varies according to the applied external magnetic field. According to [25], we can assume that the magnetic particles can be idealized as chains of particles locked in the elastomer, as shown in Figure 3. Therefore, because of the coupled magneto-elastic interactions, the shear modulus is larger under the action of a magnetic field; this can be represented by the following equations:

(4) $$\Delta G=\frac{9}{8}\frac{\Phi Cm^{2}(4-\gamma^{2})}{r_{0}^{3}\pi^{2}a^{3}\mu_{0}\mu_{‘}{\left(1+\gamma^{2}\right)}^{7/2}}$$

(5) $$m=\frac{4}{3}\pi a^{3}\mu_{0}\mu_{1}\chi H_{0}\left[\frac{1}{1-\frac{4}{3}\chi C{\left(a/r_{0}\right)}^{3}}\right]$$

(6) $$H_{0}=\frac{B}{\mu_{0}(1+\chi )}$$

where *m* is the magnetic dipole moment of the particles, a is the particle radius, χ is the susceptibility of iron particles, $\mu_{0}=4\pi \times 10^{-7}H/m$ is the absolute permeability, $\mu_{1}$ is the permeability of the MRE, $C=\sum_{j=1}^{n}\left(1/j^{3}\right)$, $r_{0}$ is the initial spacing between the two adjacent dipoles, $H_{0}$ is the intensity of the applied magnetic field, Φ is the volume fraction of particles in the MRE, and γ is the shear strain.

Because of their material properties, we assume that the MREs in the guiding mechanism are susceptible to shear forces that result in shear deformation. Owing to a structural constraint, the MREs generate the same transverse displacement as the guiding flexure beams. Thus, we have the following relationship:

(7) $$\gamma =\tan\beta =\frac{\delta }{L}$$

(8) $$F_{m}=G\gamma A_{m}=A_{m}\gamma \left(G_{0}+\Delta G\right)$$

where β is the shear deformation angle for the MREs, $A_{m}$ is the shear area, and $G_{0}$ is the initial shear modulus of the zero field.

#### 3.2.3. Modeling of the Sandwiched Guiding Mechanism

By substituting Equation (3) into Equation (7) and then substituting the resulting equation into Equation (8), it can be understood that the forces applied to the guiding flexure beams and MREs should satisfy the relationship described by Equation (9):

(9) $$F_{m}=\frac{\left(G_{0}+\Delta G\right)A_{m}L^{2}}{12EI}F_{b}$$

Correspondingly, the driving force applied to the guiding mechanism should be:

(10) $$F_{d}=4F_{b}+2F_{m}=\left(4+\frac{\left(G_{0}+\Delta G\right)A_{m}L^{2}}{6EI}\right)F_{b}$$

Note that $F_{b}=\frac{12EI}{L^{3}}\delta$ according to Equation (3). Thus, by substituting $F_{b}$ into Equation (10), we have:

(11) $$F_{d}=\left(\frac{48EI}{L^{3}}+\frac{2\left(G_{0}+\Delta G\right)A_{m}}{L}\right)\delta$$

Then, we can determine the equivalent stiffness of the guiding mechanism as a function of the external magnetic field, as follows:

(12) $$\begin{array}{cc} K_{g} & =\frac{F_{d}}{\delta }=\frac{48EI}{L^{3}}+\frac{2\left(G_{0}+\Delta G\right)A_{m}}{L}\\ & =\frac{48EI}{L^{3}}+\frac{2A_{m}}{L}\left(G_{0}+\frac{2\phi C(4-\gamma^{2})a^{3}\mu 1\chi^{2}B^{2}}{r0^{3}\mu 0(1+\chi {)}^{2}{\left(1+\gamma^{2}\right)}^{\frac{7}{2}}{\left[1-\frac{4}{3}\chi C{\left(\frac{a}{r0}\right)}^{3}\right]}^{2}}\right) \end{array}$$

From Equation (12), it is clear that the equivalent stiffness of the guiding mechanism increases with the square of the magnetic field intensity.

### 3.3. Dynamic Analysis of Compliant Mechanisms

This section describes the process of using the Lagrange method to establish a dynamic model of the compliant mechanism. Because the flexure beams and MREs are subject to damping dissipation, we introduce Rayleigh’s dissipation function $R=\frac{1}{2}c{\dot{q}}_{i}^{2}$ into Lagrange’s equation. Subsequently, Lagrange’s equation can be rewritten as:

(13) $$\frac{d}{dt}\left(\frac{\partial L}{\partial {\dot{q}}_{i}}\right)-\frac{\partial L}{\partial q_{i}}+\frac{\partial R}{\partial {\dot{q}}_{i}}=Q_{i}$$

where $q_{i}$ is the generalized coordinate, $Q_{i}$ is the generalized force without the conservative force, and L=T−V is the Lagrange term, which is the difference between the kinetic energy *T* and potential energy *V* of the system.

Next, we select the output displacement δ(t) of the central motion platform as the generalized coordinates. The kinetic energy of the entire mechanism is sourced from the movement of the central motion platform, flexure beams, and MREs, which can be described as:

(14) $$T=\frac{1}{2}M_{c}{\dot{\delta }}^{2}+4\int_{0}^{L}\frac{1}{2}\rho_{b}A_{b}{\dot{y}}^{2}dx+2\int_{0}^{L}\frac{1}{2}\rho_{m}A_{m}{\dot{h}}^{2}dx$$

where $M_{c}$ is the mass of the central motion platform, $\rho_{b}$ and $A_{b}$ are the density and cross-sectional area of the flexure beam, respectively, y is the transverse displacement at the arbitrary cross section of the flexure beam, $\rho_{m}$ and $A_{m}$ are the density and cross-sectional area of the MREs, respectively, and h is the transverse displacement at the arbitrary cross section of the MREs.

Combining Equations (2) and (3), we obtain the following relationship between y and δ:

(15) $$y=\frac{x^{2}\left(3L-2x\right)}{L^{3}}\delta$$

Taking into account the shear deformation of the MREs, we can easily derive the relationship between h and δ:

(16) $$h=\frac{x}{L}\delta$$

By substituting Equations (15) and (16) into Equation (14), we obtain the total kinetic energy as:

(17) $$T=\left(\frac{1}{2}M_{c}+\frac{26}{35}\rho_{b}A_{b}L+\frac{1}{3}\rho_{m}A_{m}L\right){\dot{\delta }}^{2}$$

The potential energy is sourced from the deformations of the flexure beams and MREs, and is given as:

(18) $$\begin{array}{cc} V & =4\int_{0}^{L}\frac{1}{2EI}\left(\frac{\partial^{2}y}{\partial x}\right)dx+2\int_{0}^{L}\frac{1}{2}\left(G_{0}+\Delta G\right)\gamma^{2}A_{m}dx\\ & =\left(\frac{24EI}{L^{3}}+\frac{\left(G_{0}+\Delta G\right)A_{m}}{L}\right)\delta^{2}=\frac{1}{2}K_{g}\delta^{2} \end{array}$$

Note that the generalized force $Q_{i}$ is the driving force $F_{vcm}$ applied to the guiding mechanism of the VCM. By substituting T, V, R, and $Q_{i}$ into Lagrange’s equation (i.e., Equation (13)), the dynamic equation for the entire micromotion platform can be expressed as:

(19) $$\left(M_{c}+\frac{52}{35}\rho_{b}A_{b}L+\frac{2}{3}\rho_{m}A_{m}L\right)\ddot{\delta }+c\dot{\delta }+K_{g}\delta =F_{vcm}$$

Correspondingly, the natural frequency $\omega_{n}$ of the compliant mechanism can be derived as:

(20) $$\begin{array}{cc} \omega_{n} & =\sqrt{\frac{K_{g}}{M_{c}+\frac{52}{35}\rho_{b}A_{b}L+\frac{2}{3}\rho_{m}A_{m}L}}\\ & =\sqrt{\frac{48EI+2A_{m}L^{2}\left(G_{0}+\frac{2\Phi C(4-\gamma^{2})a^{3}\mu_{1}\chi^{2}B^{2}}{r_{0}^{3}\mu_{0}{\left(1+\chi \right)}^{2}{\left(1+\gamma^{2}\right)}^{\frac{7}{2}}{\left(1-\frac{4}{3}\chi C{\left(\frac{a}{r0}\right)}^{3}\right)}^{2}}\right)}{M_{c}L^{3}+\frac{52}{35}\rho_{b}A_{b}L^{4}+\frac{2}{3}\rho_{m}A_{m}L^{4}}} \end{array}$$

### 3.4. Validation via Finite Element Simulations

**Figure 4:**
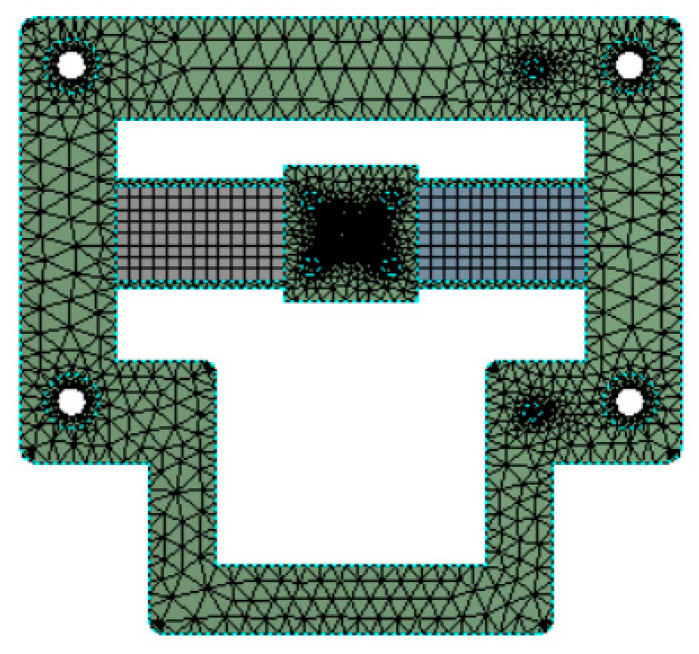
Finite element analysis model.

The critical parameters and material parameters listed in Table 1 and Table 2 were used to conduct the FEA of the proposed nanopositioner model using the ANSYS 17.0 Workbench (Canonsburg, PA, USA), as shown in Figure 4. The static and modal analyses were carried out under the conditions of different magnetic fields to verify the variable stiffness capability and dynamic tunable performance of the MRE-based nanopositioner model.

## 4. Results and Discussion

### 4.1. Stiffness Validation

**Figure 5:**
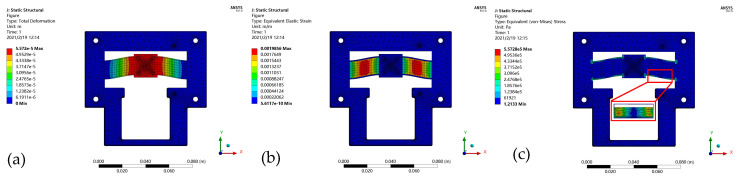
Static analysis results for the micromotion platform: (**a**) Output displacement; (**b**) Strain cloud diagram; and (**c**) Stress cloud diagram.

We first performed a static analysis to verify the variable stiffness of the developed nanopositioner model. Displacement constraints were applied to the four fixing holes, and a driving force was applied to the input surface of the central motion platform. Figure 5 demonstrated the simulation results of the deformation (Figure 5a,b) and stress distributions (Figure 5c). A large working range (2.5 mm) was enabled by the developed compliant mechanism of the nanopositioner model. 

**Figure 6:**
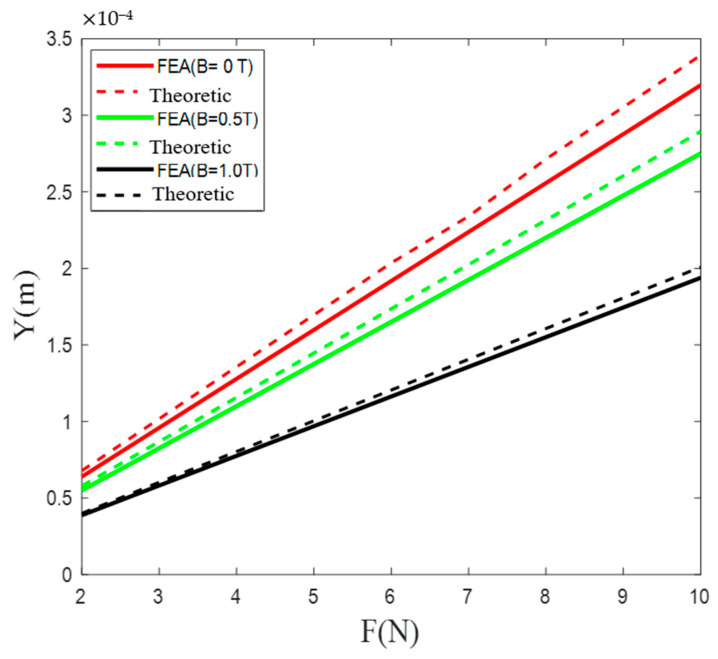
Load–displacement curves for different applied magnetic fields.

**Figure 7:**
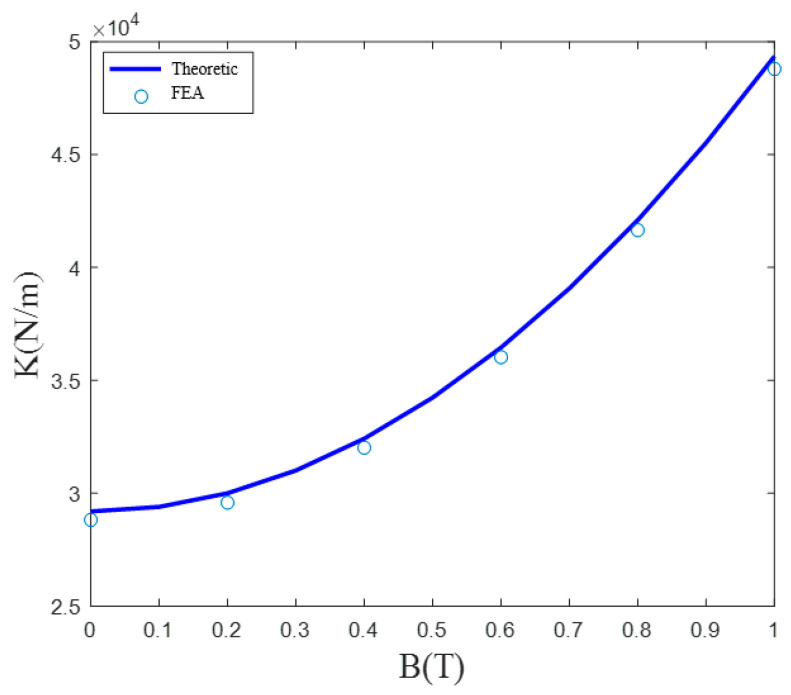
Equivalent stiffness as a function of the applied magnetic field strength.

By applying external magnetic fields with different intensities, we were able to construct the load-displacement curves illustrated in Figure 6. It is clear that the developed nanopositioner model exhibited a constant stiffness in the presence of a magnetic field with a certain intensity. As the intensity of the magnetic field increased, the equivalent stiffness of the nanopositioner model sharply increased, as indicated in Figure 7. In particular, relative to the condition of no applied magnetic field, the stiffness improved by 69.05% in the presence of a magnetic field with an intensity of 1 T. In addition, the good agreement between the theoretical results and FEA simulations demonstrates the ability of the developed static model of the nanopositioner to accurately predict the variable stiffness characteristics.

### 4.2. Dynamic Analysis

**Figure 8:**
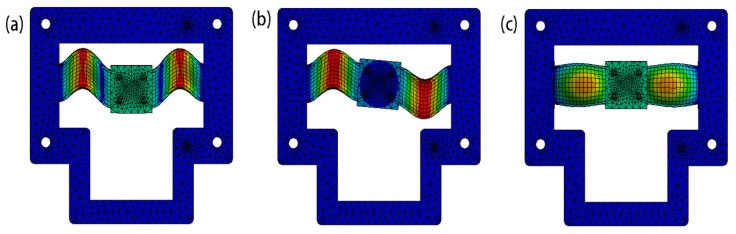
First three modes of the nanopositioner model. (**a**) 1st mode; (**b**) 2nd mode; (**c**) 3rd mode.

A modal analysis and harmonic response analysis were also conducted to verify the dynamic tunability of the developed nanopositioner model. The simulated results for the first three mode shapes, which were derived under the condition of no applied magnetic field, are illustrated in Figure 8; the corresponding frequencies are listed in Table 3. It can be seen that the first mode (Figure 8a) represents the deflection motion along the working axis and has a natural frequency of 247.58 Hz. The nanopositioner rotated around the z-axis in the second mode (Figure 8b) and translated outside the plane in the third mode (Figure 8c). 

**Figure 9:**
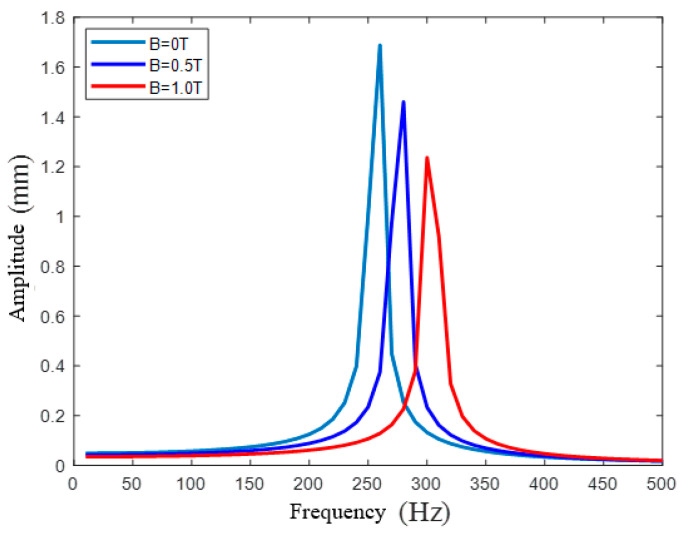
Frequency response for different magnetic fields.

The equivalent stiffness of the proposed nanopositioner model increased under the action of an external magnetic field. Correspondingly, the natural frequency, which determines the mechanical bandwidth and dynamic behavior, also significantly increased, as indicated by the frequency response results illustrated in Figure 9. Table 4 lists the natural frequencies of the nanopositioner model that were determined in the presence of various magnetic fields. Given that the discrepancy between the FEA and theoretical results was less than 4.7%, it is clear that the developed dynamic model was highly congruent with the simulated results. If the nanopositioner model is placed in a magnetic field with an intensity of 1 T, the natural frequency is expected to increase by 30.38% relative to that corresponding to an environment without a magnetic field. It is also worth mentioning that the damping performance of the nanopositioner model can also be influenced by the magnetic field because of changes in the material properties of the MREs, which were found to isolate vibrations better in the developed nanopositioner model. 

### 4.3. Experiemental Validations

**Figure 10:**
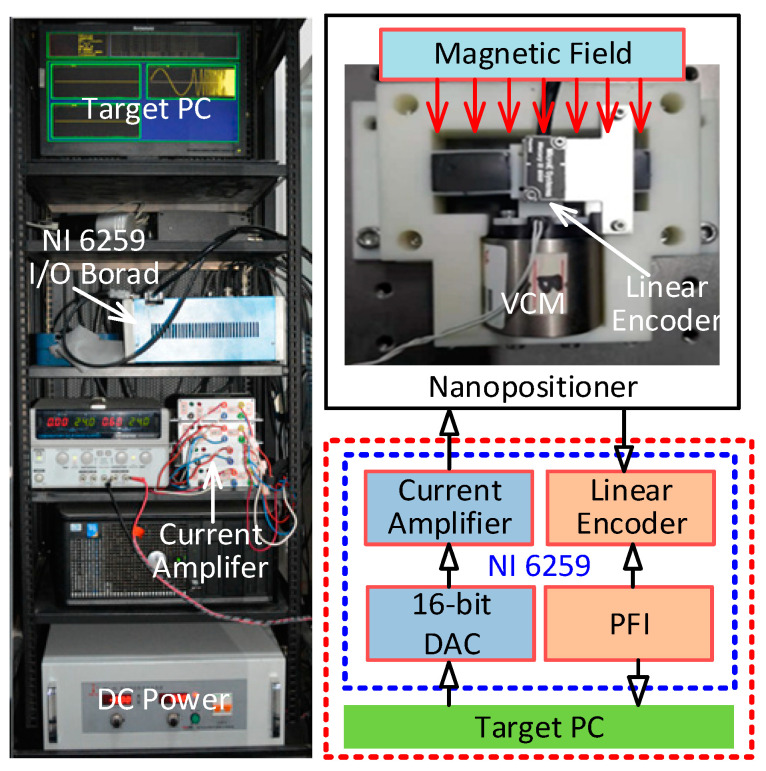
Experimental setup of the nanopositioner prototype.

Based on the structure parameters listed in Table 2, a prototype of the developed nanopositioner was machined by additive manufacturing, as illustrated in Figure 10. MREs were fabricated with carbonyl iron powder, silicone oil, and silicone rubber and were mounted inside the guiding beams. The VCM (BEI LA15-16-024) (Sensata Technologies, Attleboro, MA, USA) driven by a high bandwidth current amplifier was adopted to actuate the compliant stage. A linear encoder (Mercury II™ 6000) (Celera Motion, Bedford, MA, USA) with a resolution of 1.2 nm was used to measure the displacement information. Accordingly, an experimental system was set up through the software Matlab (R2016b, MathWorks.Inc, Natick, MA, USA) using the control package Simulink Real-Time, with National Instruments data acquisition hardware (PCI 6259) (National Instruments, Austin, TX, USA), for the purpose of test and control implementations.

**Figure 11:**
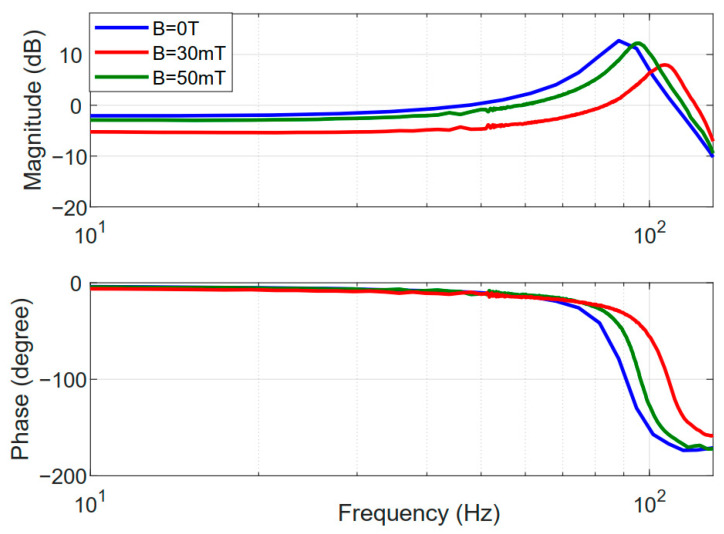
Frequency response of the nanopositioner prototype.

The dynamic-tunable performance of the developed nanopositioner was examined by the frequency response method under the action of various external magnetic fields. A swept-sine signal ranging from 1 Hz to 150 Hz was applied to the VCM, and the responding output displacements were recorded. Based on the Fourier transformation, the Bode plots of the frequency responses of the developed prototype were obtained, as shown in Figure 11. Note that the moving mass of the VCM, which is not considered in the FEA simulations, significantly affects the natural frequency of the nanopositioner, resulting in the discrepancy between the experimental and simulated results. Under the action of the external magnetic field, the natural frequency of the prototype is significantly improved, which results from the increased stiffness. On the other hand, the decrease of the system magnitude at low frequencies indicates the cost of motion range. By changing the magnetic field, the proposed nanopositioner prototype is capable of adjusting the trade-offs between the working range, speed, and sustained load capability.

## 5. Conclusions

To satisfy diverse requirements for the dynamic performance of nanopositioners, we exploited the material properties of MREs to develop a dynamically tunable nanopositioner. The elastic properties of the MREs were found to change under the action of an external magnetic field; this resulted in significant variations in the stiffness and natural frequency of the sandwiched guiding mechanism. Consequently, the proposed nanopositioner can be dynamically controlled to adjust the trade-off between the mechanical bandwidth and working range. Specifically, the developed nanopositioner can not only become more rigid when a higher positional accuracy or a larger load is required, but it can also become compliant in order to increase the working range. Theoretical models were also established to predict the variable stiffness characteristics and dynamically tunable properties of the nanopositioner; these models were validated by performing FEA. The noncontact tuning method, which uses an external magnetic field, has the potential to be used in various extreme applications. In the future, we plan to conduct experimental studies using the developed nanopositioner.

## Figures and Tables

**Table 1:** Material parameters of the nanopositioner.

Parameter	Symbol	Value
Young’s modulus of flexure beams	*E* (GPa)	2.2
Poisson’s ratio of flexure beams	*μ*	0.39
Density of flexure beams	*ρ_m_* (kg/m^3^)	1190
Initial shear modulus of magnetorheological elastomers (MREs)	*G*_0_ (MPa)	0.8
Permeability of MREs	*μ*_1_ (H/m)	3.5
Density of MREs	*ρ_b_* (kg/m^3^)	1100

**Table 2:** Geometric parameters of the nanopositioner.

Parameter	Symbol	Value
Length of flexure beams	*L* (mm)	25
Width of flexure beams	*b_b_* (mm)	20
Thickness of flexure beams	*t_b_* (mm)	1
Width of MREs	*b_m_* (mm)	14
Thickness of MREs	*t_m_* (mm)	20
Cross-sectional area of the MREs	*A_m_* (mm^2^)	280
Length of central motion platform	*l_c_* (mm)	20
Width of central motion platform	*w_c_* (mm)	20
Height of central motion platform	*h_c_* (mm)	20

**Table 3:** ANSYS results for the frequency modes in the absence of an applied magnetic field.

Modes	1st	2nd	3rd
Frequency (Hz)	247.58	909.31	958.87

**Table 4:** Natural frequency under different magnetic fields.

Magnetic Field Intensity	B = 0 T	B = 0.5 T	B = 1.0 T
Theoretical	236.46	259.89	315.73
FEA	247.58	262.95	316.05
